# Ring-augmented versus conventional one-anastomosis gastric bypass: Perioperative and short-term results in the randomized controlled RiMini trial

**DOI:** 10.1371/journal.pone.0349793

**Published:** 2026-06-01

**Authors:** Maureen Tissink, Josien Timmerman, Marloes Vermeer, Tim Verhagen, Ian Faneyte, Eric Hazebroek, Marc van Det

**Affiliations:** 1 Hospital Group Twente Almelo, Netherlands; 2 Wageningen University, Wageningen, Netherlands; 3 Rijnstate Hospital, Arnhem, Netherlands; Shahid Beheshti University of Medical Sciences School of Medicine, IRAN, ISLAMIC REPUBLIC OF

## Abstract

**Purpose:**

Recurrent weight gain remains a challenge in metabolic bariatric surgery (MBS). Recent publications focused on ring-augmented Roux-en-Y gastric bypass (RYGB) and ring-augmented sleeve gastrectomy (SG), but few studies have addressed the potential of ring-augmentation for one-anastomosis gastric bypass (OAGB).

**Objectives:**

The RiMini trial is a single-center randomized controlled trial that investigates the difference in long-term weight reduction, associated medical comorbidities, quality of life, and procedure-related adverse events of ring-augmented OAGB compared to conventional OAGB in adult patients eligible for primary OAGB. This analysis reports the trial’s short-term outcomes, including the peri-operative safety of the procedure, short-term adverse events, and weight outcomes at one year postoperatively.

**Methods:**

Between July 2022 and December 2023, a total of 214 patients (107 per group) underwent either ring-augmented or conventional OAGB after randomization. Peri- and postoperative adverse events, and total and excess weight loss percentages (%TWL and %EWL) were assessed by intention-to-treat and per-protocol analysis.

**Results:**

At baseline, there were no differences between groups regarding age, gender, and body mass index (BMI). Mean operation time was 51 minutes (±13) in both groups (p = 0.84). One patient in each group experienced a perioperative complication (p = 1.00; 0.9%). There was no significant difference in postoperative minor (Clavien Dindo (CD) 1–2) (p = 0.68) or major (CD3–5) (p = 0.77) complications. In the first year, two Minimizer rings were electively removed at patients’ request, without observed complications related to the ring. At one year, mean BMI was comparable in both groups (28 kg/m^2^). There was no statistically significant difference between ring-augmented and conventional OAGB in %TWL (33% vs 31%; p = 0.30), and %EWL (86% vs 84%; p = 0.45).

**Conclusions:**

The 1-year analyses of the RiMini trial showed that ring-augmented OAGB is comparable in safety to conventional OAGB. At 1 year, there was no statistically significant difference in weight loss between the groups.

## Introduction

Obesity is increasingly recognized as a major global health concern. The latest data from the World Health Organization (WHO) shows that one in eight people worldwide live with obesity (BMI ≥ 30 kg/m^2^) [1]. This represents a doubling of the number of individuals with obesity compared to 1990, and prognoses indicate that this number will continue to rise in the upcoming years. Obesity is associated with a wide range of associated medical problems, including type 2 diabetes, cardiovascular diseases, obstructive sleep apnea, and certain types of cancer. Beyond the individual health burden, obesity poses substantial economic costs on healthcare systems and society due to increased medical expenses and loss of productivity. Metabolic bariatric surgery (MBS) remains the most effective treatment for achieving optimal initial clinical response and reducing or remitting associated medical problems [2].

Worldwide the sleeve gastrectomy (SG) is the most commonly performed bariatric procedure (55.4%), followed by the Roux-en-Y gastric bypass (RYGB; 29.3%) and the one-anastomosis gastric bypass (OAGB; 6.6%). The OAGB continues to rise in popularity worldwide [3].

Approximately 30% of patients will regain more than 25% of their total weight loss within two to five years after MBS [4]. Recurrent weight gain after MBS is not only associated with lifestyle and behavioral factors, but also with anatomical changes such as dilation of the pouch, widening of the gastrojejunal anastomosis, or expansion of the proximal jejunum. To limit these anatomical changes and reduce recurrent weight gain, the addition of a non-adjustable silicone ring around the reduced stomach has been introduced as an adjunct to MBS. While multiple studies have demonstrated that adding a ring to sleeve gastrectomy (SG) or Roux-en-Y gastric bypass (RYGB) is safe and may improve long-term weight stability [5,6], it remains unclear whether similar benefits can be achieved with ring augmentation in one-anastomosis gastric bypass (OAGB). Moreover, the safety, feasibility, and overall effectiveness of ring-augmentation in primary OAGB have not yet been thoroughly assessed.

The aim of the RiMini trial is to investigate whether augmentation with a silicone ring during primary OAGB improves weight loss outcomes and prevents recurrent weight gain, associated medical problems, quality of life, and is not associated with an increased rate of procedure-related adverse events compared to conventional OAGB [7]. This article presents an interim analysis of the RiMini trial, focusing solely on short-term safety and feasibility of ring-augmented versus conventional OAGB, including peri- and postoperative adverse events and early weight-loss outcomes during the first year of follow-up.

## Methods

### Study design

The RiMini trial is a single-center, non-blinded, prospective randomized controlled trial. This study is registered at Clinicaltrials.gov (NCT05472922) on the 25th of July 2022. This study was approved by the Medical research Ethics Committees United (MEC-U). Patients were screened and accepted for primary MBS based on the guidelines of the International Federation for the Surgery of Obesity and Metabolic Disorders (IFSO) [2]. The decision to undergo OAGB was made by the patient in consultation with the treating bariatric surgeon. After providing written informed consent, eligible patients were randomized in a 1:1 ratio to receive either ring-augmented or conventional OAGB.

### Surgical procedures

For the OAGB, a long and narrow gastric pouch is formed from the angle of the lesser curvature to the angle of His. A standard-length biliary limb of 150 centimeters for patients with BMI up to 50 kg/m^2^ or 180 centimeters for patients with BMI above 50 kg/m^2^ is drawn cranially in an antecolic antegastric position. In the intervention group, a Minimizer Ring (Bariatric Solutions GMBH, CH-8260 Stein am Rhein) was placed around the gastric pouch two to four centimeters below the esophagogastric junction after completion of the conventional OAGB. The inclusion period and surgical interventions ran from July 2022 to December 2023. All patients received multidisciplinary pre- and postoperative care according to the local standard care, including ‘Modifast Intensive®’ diet in the two weeks prior to surgery. Further methodological details of the Rimini trial can be found in the recently published study protocol (DOI: 10.1007/s11695-025-07751-6) [7].

### Outcomes

In order to assess the short-term safety of the procedure, including peri- and postoperative complications and adverse events within 12 months, the early results were analyzed. Weight-loss parameters were evaluated as secondary outcomes. Sample size of this analysis was based on the primary endpoint of the RiMini trial: weight loss at five years. Assuming a sample of 63 individuals per group and an expected loss to follow-up of 40%, a total of 210 patients (105 per group) were required.

Other prespecified secondary outcomes of the RiMini trial, including changes in associated medical comorbidities and quality of life, were collected at predefined follow-up intervals (4 months and 1 year), but are not reported in the present manuscript and will be analyzed separately in accordance with the trial protocol [7].

### Outcome measurements

To evaluate safety, all complications were systematically recorded and analyzed. Complications were assessed and scored according the Clavien-Dindo (CD) scale and classified as ‘minor’ when no treatment or medical treatment was needed (CD 1–2), and as ‘major’ if surgical re-intervention or therapeutic endoscopy was necessary and/or treatment on an intensive care unit was needed (CD 3–4) [8]. Complications were categorized as perioperative, early postoperative (< 30 days), or late postoperative (≥ 30 days). For each complication in the intervention group, a potential association with the ring was investigated.

To assess the clinical impact of adding a ring to the OAGB procedure, the operative time and the length of hospital stay, were analyzed and compared between the two groups. A proportion of patients were treated in a day-care setting and discharged on the day of surgery.

To analyze the effect of ring-augmentation in OAGB, both total (%TWL) and excess (%EWL) weight loss was calculated using body weight and BMI measured on the day of surgery and at predefined follow-up intervals (4 months and 1 year) for both groups. This deviates from the original protocol, which proposed the date of informed consent as the baseline. Due to substantial variation between consent and surgery dates, the day of surgery was chosen as a standardized baseline to ensure consistency across patients [7].

### Statistical analysis

Descriptive statistics were used to summarize baseline characteristics and outcomes. Continuous variables were reported as means ± standard deviations or medians with interquartile ranges, depending on their distribution.

Normality was assessed through visual inspection of histograms inspection. Categorical variables were reported as number with corresponding percentage.

Differences in baseline characteristics and outcomes between the groups were tested using the independent t-test (for continuous, normally distributed data), Mann-Whitney U test (for non-normally distributed or ordinal data), and the Chi-square or Fisher’s exact test (for categorical data), as appropriate.

Where applicable, linear regression models were used to adjust for potential confounders identified at baseline (p < 0.15). A two-sided p-value < 0.05 was considered statistically significant.

To assess whether vari‌‌ation in the timing of the one-year follow-up influenced the postoperative weight-loss outcomes, a Pearson correlation analysis was performed between the number of days from surgery to the one-year follow-up visit and the corresponding weight-loss percentages.

Primary outcome analyses of this manuscript followed the intention-to-treat (ITT) principle, with additional per-protocol (PP) analyses performed for completeness and transparency. The per-protocol population consisted of all patients who underwent the allocated procedure and completed follow-up without major protocol deviations [7]. Patients who required intraoperative conversion or postoperative ring removal were excluded from the per-protocol analysis but remained included in the intention-to-treat analysis. For this interim analysis, missing values were not imputed.

All analyses were performed using R version 4.2.2.

## Results

### Baseline patient characteristics

A total of 218 patients were recruited in the RiMini trial, with each group containing 109 patients. A total of 2 patients per group were found to be unfit for OAGB perioperatively (see Appendix 1, perioperative results). All reported baseline characteristics and subsequent outcomes therefore reflect the 214 patients who underwent the intended surgical procedure.

There were no significant differences in patient demographics and their associated medical comorbidities at baseline (Table 1). The mean age of both groups was 43 years (±11). The ring-augmented OAGB group (intervention) included 85 female patients (79%). The mean BMI on the day of surgery was 41.6 kg/m^2^ (±6.1). The conventional OAGB group (control) included 80 female patients (75%). Mean time between screening and day of surgery was 60 days (±33.7) in both groups (*p = 0.75*). Mean BMI on day of surgery was 41.6 kg/m^2^ (±5.1). Both mean weight and BMI differed significantly between the screening and the day of operation in both groups (*p < 0.001*). As shown in Table 1, no significant differences were observed between the two groups in BMI or mean weight at screening or at the day of operation. Furthermore, the interval between screening and surgery and the extent of preoperative weight change were comparable between groups.

**Table 1:** Baseline characteristics.

	Total (N = 214)	Ring-augmented (N = 107)	Conventional (N = 107)	P value
Sex				0.51
*Female, n (%)*	165 (77)	85 (79)	80 (75)	
*Male, n (%)*	49 (23)	22 (21)	27 (25)	
Age in years, mean (SD)	43 (±11)	43 (±11)	43 (±11)	0.97
Weight in kg, mean (SD)				0.50 0.97
*Screening*	128.6 (±18.6)	127.8 (±17.4)	129.5 (±19.7)	
*Operation day*	124.3 (±17.7)	124.2 (±18.0)	124.3 (±17.6)	
BMI in kg/m^2^, mean (SD)				0.49 0.97
*Screening*	43.1 (±5.6)	42.8 (±5.8)	43.3 (± 5.5)	
*Operation day*	41.6 (±5.6)	41.6 (±6.1)	41.6 (± 5.1)	
				
Diabetes Mellitus, n (%)	30 (14.0)	17 (15.9)	13 (12.1)	0.19
*Type 1*	2 (0.9)	0	2 (0.9)	
*Type 2*	28 (14.0)	17 (15.9)	11 (10.3)	
*Medication, n (%)*	25 (11.7)	14 (13.1)	11 (10.3)	0.67
Hypertension, n (%)	63 (29.4)	30 (28.0)	33 (30.1)	0.76
*Medication, n (%)*	57 (26.6)	28 (26.3)	29 (27.1)	1.00
Dyslipidemia, n (%)	36 (16.8)	17 (15.9)	19 (17.8)	0.86
*Medication, n (%)*	26 (12.1)	13 (12.1)	13 (12.1)	1.00
Osteoarthrosis, n (%)	52 (24.2)	26 (24.3)	26 (24.3)	1.00
OSAS, n (%)	57 (26.6)	25 (23.4)	32 (29.9)	0.35
*CPAP, n (%)*	42 (19.6)	20 (18.7)	22 (20.6)	0.86
GERD, n (%)	40 (18.7)	18 (16.8)	22 (20.6)	0.60
*Medication, n (%)*	31 (14.5)	12 (11.2)	19 (17.8)	0.24

***OSAS****, Obstructive Sleep Apnea Syndrome;* ***CPAP****, continues positive airway pressure;* ***GERD****, Gastro Esophageal Reflux Disease;* ***SD****, standard deviation.*

The mean interval between the day of surgery and the first follow-up visit (4 months) was 130 days (±15), and 382 days (±28) for the second follow-up visit (1 year). These timeframes deviated slightly from the study protocol, which listed the planned follow-up points but did not define an acceptable window of deviation around those time points [7].

**Fig 1:**
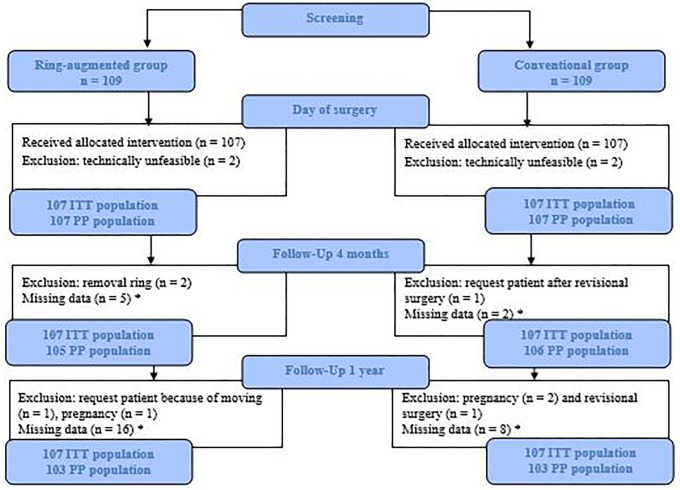
Flowchart in- and exclusion and missing data per timepoint following ITT and PP principle. ITT Intention-to-treat. PP Per protocol. *Missing data due to no show of patient or scheduling errors.

For each timepoint, the reasons for exclusion and missing data were described following ITT and PP analyses (Fig 1). Missing data were not classified as ‘lost to follow-up’, as follow-up completeness varied by time point and the trial is still ongoing. Therefore, each time point, missing data were described and reasons were provided when known.

### Perioperative results

All included patients in the study were randomized and underwent surgery with the intention to perform an OAGB. Four patients did not receive OAGB, two in both groups, due to limited visibility caused by adhesions or technical problems with pouch creation, and were converted to either a RYGB or SG. These patients were excluded from both the ITT- and PP-analysis in this study (Fig 1). For the remaining 214 patients, OAGB was successfully performed.

#### Perioperative outcomes.

The mean procedure time (skin to skin) was 51 minutes (±13) in both groups (p = 0.84). Placement of the silicone ring was successful in all patients in the intervention group and did not result in perioperative complications or statistically significantly longer procedure times.

#### Postoperative outcomes.

Mean length of hospital stay was 0.82 days (±0.56) in the intervention group and 1.08 days (±1.41) in the control group, both with a median of 1 day (p = 0.08).

Because a proportion of patients was treated in a day-care setting and discharged on the day of surgery, length of stay was expressed in fractional days, resulting in mean values below 1 day.

### Adverse events and complications

During the first year of the follow-up of the RiMini trial, a total of 17/214 patients (8%) experienced one or more peri- or postoperative adverse events (Table 2): 11/107 patients (10%) in the intervention group and 6/107 patients (5.6%) in the control group. Absolute rates did not statistically significantly differ between the two groups (*p = 0.5*). For full transparency, absolute numbers of complications are reported based on the intention-to-treat population (n = 107 per group).

**Table 2:** Number of perioperative adverse events, and early and late postoperative complications.

	Ring-augmented	Conventional	P value*
**Perioperative adverse event**	1	1	1.00
**Early postoperative complication**			
*Minor (CD 1–2)*	2	2	1.00
*Major (CD 3–4)*	4	2**	0.68
**Late postoperative complication**			
*Minor (CD 1–2)*	2	0	0.50
*Major (CD 3–4)*	2	2**	1.00

***CD*** Clavien Dindo. *Fisher’s exact test. **Two complications in one patient (early and late).

#### Perioperative adverse events.

One patient (0.9%) in each group experienced a minor perioperative adverse event: significant blood loss (> 300cc) due to iatrogenic injury. Both patients recovered well without blood transfusion, re-operation or prolonged hospital admission.

#### Early postoperative complications (< 30 days).

Four patients experienced early **minor** complications (CD 1–2): two patients in each group (1.9%). In the ring-augmented group, one patient experienced abdominal pain including a rise in infection parameters pragmatically treated with intravenous antibiotics (n = 1). One patient experienced difficulty with oral intake (n = 1) requiring hospital admission. In the conventional group, complications involved difficulties with oral intake (n = 1) requiring hospital admission and a blood transfusion due to hemoglobin decline (n = 1). All patients recovered without further intervention.

Six patients experienced early **major** complications (CD 3–4): four (3.7%) in the ring-augmented group and two (1.9%) in the conventional group (p = 0.68) In the ring-augmented group, these included staple line dehiscence, biliopancreatic limb obstruction, intra-abdominal hematoma, and intra-abdominal abscess, all requiring laparoscopic re-intervention. In the latter case, although no ring-related issue was found during re-laparoscopy, the patient requested ring removal and was subsequently withdrawn from the following study analyses. In the control group, one patient developed an intra-abdominal hematoma and another patient a blowout of the remnant stomach, both treated laparoscopically.

#### Late postoperative adverse events (≥ 30 days).

Two patients (1.9%) in the ring-augmented group developed late **minor** adverse events, characterized by abdominal pain without an identifiable cause, which resolved spontaneously following clinical observation.

A total of four patients developed late **major** adverse events: two in both groups (1.9%). In the ring-augmented group, one patient had persistent impaired oral intake despite normal findings; after ring removal at three months on patients request and subsequent conversion to RYGB at seven months, symptoms persisted. Another patient developed a perforated marginal ulcer that was oversewn laparoscopically. In the conventional group, one patient underwent conversion to RYGB for therapy-resistant GERD, and one patient for recurrent bleeding from a marginal ulcer.

A detailed description of major complications and adverse events can be found in Appendix 1.

#### Ring-related adverse events.

No direct causal link to the Minimizer ring was identified in any of the patients with adverse events, either during surgery or through objective additional investigations such as contrast swallow study or gastroscopy. Two Minimizer rings were removed on patients’ request as mentioned above (Appendix 1).

### Weight Loss

At both predefined follow-up intervals (4 months and 1 year), ITT and PP analyses showed comparable results regarding BMI (see Table 3).

**Table 3:** BMI in kg/m^2^ after 4 months and 1 year.

	Ring-augmented		Conventional		
ITT-analysis	Mean (±SD)	N	Mean (±SD)	N	P value*
4 months	33.4 (4.0)	102/107	33.6 (4.8)	105/107	0.75
1 year	28.1 (5.0)	91/107	28.3 (4.2)	99/107	0.76
PP-analysis	**Mean (±SD)**	**N**	**Mean (±SD)**	**N**	P value*
4 months	33.4 (5.3)	100/105	33.6 (4.8)	104/106	0.78
1 year	28.0 (5.0)	87/103	28.2 (4.2)	95/103	0.77

*Number of patients based on available data at follow-up points for both analyses (* Fig 1 *).*

***BMI*** *Body Mass Index.* ***SD*** *Standard Deviation.* ***P value*** calculated with *unpaired t-test. ***ITT*** *Intention-to-treat.* ***PP*** *Per protocol.*

As shown in Table 4, there were no statistically significant differences in %TWL and %EWL at four months and one year between ring-augmented OAGB and conventional OAGB in both the ITT- and the PP-analyses. Despite variability in the actual timing of follow-up visits, Pearson correlation analysis showed no significant correlation between the number of days from surgery to follow-up and the observed weight-loss percentages (*r = −0.017, p = 0.823*).

**Table 4:** %TWL and %EWL after 4 months and 1 year.

	Ring-augmented		Conventional		
ITT-analysis	Mean (±SD)	N	Mean (±SD)	N	P value*
%TWL 4 months	19.93 (±4.0)	102/107	19.49 (±4.4)	105/107	0.44
%EWL 4 months	53.46 (±16.4)	102/107	51.96 (±17.2)	105/107	0.52
%TWL 1 year	32.71 (±7.3)	91/107	31.87 (±7.3)	99/107	0.43
%EWL 1 year	85.86 (±22.8)	91/107	83.86 (±23.1)	99/107	0.55
					
PP-analysis	**Mean (±SD)**	**N**	**Mean (±SD)**	**N**	P value*
%TWL 4 months	19.92 (±4.0)	100/105	19.49 (±4.4)	104/106	0.46
%EWL 4 months	53.13 (±15.6)	100/105	51.96 (±17.2)	104/106	0.61
%TWL 1 year	33.02 (±6.9)	87/103	31.91 (± 7.4)	95/103	0.30
%EWL 1 year	86.85 (±22.3)	87/103	84.34 (± 23.0)	95/103	0.45

*Number of patients based on available data at follow-up points for both analyses (* Fig 1 *).*

***%TWL*** *Percentage total weight loss.* ***%EWL*** *Percentage excess weight loss.* ***SD*** *Standard Deviation.* ***P value*** calculated with *unpaired t-test. ***ITT*** *Intention-to-treat.* ***PP*** *Per protocol.*

## Discussion

This study analyzed the peri- and postoperative outcomes, and one-year results of the prospective cohort of 214 patients who underwent OAGB in the randomized controlled RiMini trial comparing ring-augmented and conventional OAGB in primary MBS.

Baseline characteristics of the two groups were comparable. In both groups, mean weight and BMI were lower on the day of surgery than at screening, which is likely explained by the routinely prescribed preoperative Modifast diet promoting weight loss and liver volume reduction [9]. Chinaca et al showed no conclusive evidence that preoperative weight loss confers improved postoperative outcomes [10]. Therefore, the observed difference in weight and BMI between screening and day of surgery is not expected to affect postoperative outcomes. Moreover, both the interval between screening and day of surgery and the extent of preoperative weight change were comparable between groups, suggesting that the protocol deviation did not differentially affect either study arm.

In the perioperative phase, there were no statistically significant differences between the groups in operation time. Perioperative complications were minimal and had no consequences for the patients and their postoperative recovery. This implies that ring-augmented OAGB is safe and feasible for primary MBS and does not lead to a significant increase in operation time compared to conventional OAGB.

As shown earlier, there was a trend toward a statistically significant difference in mean hospital stay between both groups (p = 0.08) in favor of the ring-augmented cohort. This finding could be explained by an outlier in the control group, where one patient required prolonged postoperative admission of 14 days due to a complication. However, the median hospital stay did not differ between the two groups (1 day).

Although the overall count of adverse events in the first year was higher in the intervention group (10%) compared to the conventional OAGB (5.6%), this difference was not statistically significant (*p = 0.5*). When considering the overall adverse event rates within one year, minor events occurred in 4 patients in the ring-augmented group versus 2 in the conventional group, and major events in 6 versus 4 patients, respectively.

These differences were not statistically significant (minor: p = 0.68; major: p = 0.77), reflecting the low absolute event numbers. Importantly, the incidence of clinically relevant major complications (CD 3–4) in the ring-augmented cohort is comparable to the conventional OAGB group and to other non-randomized publications with complication rates between 2.6% and 7% [11,12]. These findings should be interpreted in the context of an early safety evaluation. Long-term follow-up of the Rimini trial is required to assess the true incidence of ring-related symptoms, such as dysphagia, and to allow comparison with long-term data of other studies.

The strict follow-up and adverse event registration in this prospective randomized trial cohort might lead to more accurate registration of especially the minor complications compared to non-randomized and/or retrospective cohorts. It is worth mentioning that none of the perioperative complications or adverse events in the first year were clearly attributable to the silicone ring, supporting its safety in OAGB procedures. These findings confirm that ring-augmented OAGB is comparable to ring-augmented RYGB and SG, indicating that ring augmentation is generally safe across different bariatric procedures [6, 13,14].

At one year, ring augmentation in OAGB did not result in significantly greater weight loss. This is in line with the results of the Bandolera trial (RCT) comparing ring-augmented versus conversional RYGB [13]. Although some non-randomized cohort studies show improved weight loss in BMI at one year in their ring-augmented groups, our findings showed no early signal of benefit [12]. Compared to RYGB, also with conventional OAGB, weight loss in the first year is more rapid and more substantial [15]. In the ring-augmented group, weight loss (%TWL 33% and %EWL 87%) was comparable to other non-randomized ring-augmented OAGB cohorts, although techniques for OAGB and ring-augmentation in these cohorts had some differences [11,16]. The similar weight-loss results in both arms of this study might suggest that the restrictive effect of a ring may not yet be apparent in the first year after OAGB. Available data derived from small cohorts of ring-augmented OAGB, shows varying outcomes The absence of statistically significant differences in weight loss outcomes in the present study should therefore be interpreted with caution, as the sample size of the RiMini trial was calculated based on the primary endpoint at five-year follow-up [7]. Consequently, the study may be underpowered to detect small differences between groups at this early one-year time point. Further follow-up of our cohort will demonstrate whether ring-augmented OAGB will lead to more weight loss over time and a smaller long-term risk of recurrent weight gain.

Although follow-up visits were not performed at identical time points, no significant correlation was observed between the interval from surgery to follow-up and weight-loss outcomes, suggesting that minor variability in follow-up timing did not influence the results.

This study does have some limitations. Patients were not blinded for treatment allocation, as blinding was considered unnecessary for the quality of the study and considered potentially unsafe in case of an emergency that would have to be treated in a setting where no medical history of the patient was available. The overall complication rate observed in the intervention group was slightly higher than reported in the literature, without a clear explanation for this finding. In our analysis, no direct causal link with the Minimizer ring was identified. This discrepancy does not reflect the overall complication rate within our institution, which is reviewed annually and consistently remains within the expected benchmark range. While a proportion of patients missed the predefined 1-year follow-up window, they cannot yet be considered permanently lost to follow-up given the ongoing nature of the study.

To minimize potential bias from post–1-year weight nadir measurements, only data within the predefined timeframe were analyzed; sensitivity and imputation analyses will be performed in the planned long-term analyses if loss to follow-up exceeds anticipated rates.

## Conclusion

The one-year analysis of the RiMini trial provides substantial evidence that ring-augmented OAGB is comparable in safety to conventional OAGB. No early benefit of ring-augmented OAGB was observed in terms of %TWL or %EWL at 4 months and 1 year postoperatively compared with conventional OAGB. Long term outcomes of this study (5 years follow-up) will be crucial in determining whether ring-augmentation of OAGB will lead to improved long term weight loss and less recurrent weight gain compared to conventional OAGB.

### Informed consent

Written informed consent was obtained from all individual participants included in the study.

### Trial registration

This study is registered at Clinicaltrials.gov (NCT05472922) on the 25th of July, 2022.

### Key points

- Ring-augmented OAGB is comparable in safety to conventional OAGB;
- Ring augmentation in OAGB does not lead to a significant increase in perioperative or postoperative complications;
- After one year, there is no statistically significant difference in weight loss outcomes between ring-augmented and conventional OAGB.

## Supporting information

S1 Appendix Detailed description of perioperative and postoperative outcomes. (DOCX)

S1 File CONSORT Checklist. (DOCX)
