# Supplementary material for: Ring-augmented versus conventional one-anastomosis gastric bypass: Perioperative and short-term results in the randomized controlled RiMini trial
Source: PLoS One. 2026 Jun 1;21(6):e0349793. doi: 10.1371/journal.pone.0349793 (PMC13225348; doi:10.1371/journal.pone.0349793)
Supplement: S1 Appendix — (DOCX) [file pone.0349793.s001.docx]

**APPENDIX 1**

**Perioperative results**

Four patients did not receive OAGB, two in both groups. In the intervention group, one patient was converted to a SG due to limited visibility caused by adhesions, making it technically unfeasible to perform OAGB. In another patient perioperative assessment revealed a stapling problem, resulting in an insufficient and non-patent anastomosis. Consequently, the procedure was converted to a RYGB. In the control group, one patient was converted to SG due to extensive small bowel adhesions from prior surgery. another patient was converted to RYGB due to technical problems with pouch creation.

**Complications**

Six patients experienced **major** complications in the perioperative period: four in the intervention group and two in the control group. In the intervention group, the following complications were observed:

1. Staple line dehiscence at the angle of His on postoperative day (POD) 3. Treated with re-laparoscopy, drainage, and suturing of the defect with an omental plasty.
2. Intra-abdominal abscess with suspicion of minor staple line dehiscence at the angle of His on POD 7. Re-laparoscopy was performed; methylene blue test did not confirm a leak. The staple line was reinforced with sutures and an omental plasty. Despite no intraoperative evidence of ring-related complications, the patient requested ring removal and withdrew from the study, and was excluded from per-protocol analysis.
3. Biliopancreatic limb obstruction due to kinking at the gastrojejunostomy on POD 5. Managed with re-laparoscopy and fixation of the gastrojejunostomy.
4. Intra-abdominal hematoma on POD 2 requiring transfusion. Re-admission on POD 19 due to an infected hematoma, with re-intervention revealing staple line dehiscence at the gastrojejunostomy and residual stomach.

In the control group, the following complications were observed:

1. Intra-abdominal hematoma on POD 2, requiring laparoscopic re-intervention for evacuation.
2. Blowout of the remnant stomach on POD 4, requiring laparoscopic resection of the remnant stomach.

A total of four patients developed late **major** adverse events: two in the intervention group, and three in the control group. In the intervention group, the following complications were observed:

1. Persistent impaired oral intake, with normal findings on endoscopy and swallow studies. The patient requested ring removal at three months; however, symptoms persisted. A conversion to RYGB was performed after seven months, but symptoms remained. Due to the conversion, this patient was excluded from the per-protocol analysis.
2. Perforated marginal ulcer at the posterior side of the pouch at ten months postoperatively. The perforation was treated with oversewing and omental plasty.

In the control group the following adverse events were observed:

1. Therapy-resistant gastroesophageal reflux disease (GERD), necessitating conversion to RYGB. Patient was excluded from per-protocol analysis.
2. Recurrent bleeding from a persistent marginal ulcer, requiring conversion to RYGB. Due to conversion and on patient’s request, this patient was excluded from the per-protocol analysis. This is the same patient as mentioned under early complication 6.
